# Critical‐Network Lesion‐Symptom Mapping

**DOI:** 10.1002/hbm.70644

**Published:** 2026-09-14

**Authors:** Grant M. Walker, G. Lynn Kurteff, Akbar Hussain, Chris Rorden, Leonardo Bonilha, Julius Fridriksson, Gregory Hickok

**Affiliations:** ^1^ Department of Cognitive Sciences University of California Irvine California USA; ^2^ Department of Communication Sciences and Disorders University of South Carolina Columbia South Carolina USA; ^3^ Department of Neurology University of South Carolina Columbia South Carolina USA; ^4^ Department of Language Sciences University of California Irvine California USA

**Keywords:** aphasia, atlas‐based analysis, ensemble modeling, lesion‐symptom mapping, neuroimaging, stroke

## Abstract

Complex datasets used for lesion‐symptom mapping (LSM) present serious challenges for interpretable statistical analysis. We introduce Critical Network Lesion‐Symptom Mapping (CN‐LSM), a neuroanatomical atlas‐based method that uses low‐dimensional regression models and out‐of‐sample prediction to identify brain regions that contribute significantly to the prediction of clinical behavioral scores. Rather than focusing on a single most‐predictive model, CN‐LSM identifies a set of plausible models analyzed as an ensemble. CN‐LSM offers three primary advantages over existing approaches: (1) greater robustness to analytical decisions that should be (but often are not) inconsequential to results; (2) holistic quantification of uncertainty across the entire statistical map rather than at individual voxels or regions; and (3) explicit accommodation of multiple plausible explanations, reducing false negatives while preserving stringent control over false positive rates.

## Graphical abstract

We introduce Critical‐Network Lesion‐Symptom Mapping (CN‐LSM), an atlas‐based approach that uses ensemble modeling and out‐of‐sample prediction to identify brain networks supporting behavior in stroke patients. CN‐LSM outperforms standard multivariate methods in ground‐truth recovery while delivering interpretable, low‐dimensional results with fewer researcher degrees of freedom.

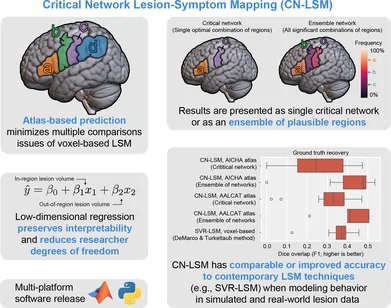

Examining the consequences of brain injury has lent great insight into functional neuroanatomy. With the advent of modern neuroimaging, a statistically grounded approach for relating damaged brain tissue to behavioral impairments emerged, called voxel‐based lesion‐symptom (or lesion‐behavior) mapping (VLSM) (Bates et al. 2003), inspired by statistical parametric mapping (Friston et al. 1994) used for functional imaging analysis. While popular, the initial use of mass univariate analyses (i.e., examining each voxel independently) lacks the ability to account for complex interrelations among brain areas, giving rise to multivariate analysis approaches such as support vector regression‐based LSM (SVR‐LSM) (Mah et al. 2014; Zhang et al. 2014). However, subsequent work has not demonstrated a universal advantage of multivariate methods over mass‐univariate approaches (Ivanova et al. 2021; Sperber and Umarova 2024). Instead, these approaches may serve complementary purposes: mass‐univariate methods offer clearer interpretability, whereas multivariate methods can exploit complex but less transparent patterns to yield more accurate predictions (Moore et al. 2024). Some of the methodological concerns that continue to hamper both of these common LSM approaches include:

1. Inference procedures based on null hypothesis statistical testing (NHST) rather than prediction‐based model comparison can distort the strength and implications of the evidence.
2. Focusing on a single, optimally predictive model (or voxel) rather than the set of plausible models can conceal important relationships.
3. Various methods for controlling nuisance covariates, particularly lesion volume, may remove important signals from the data.
4. Despite published guidelines and tutorials, there remains a potentially daunting number of decisions to be made that will influence the outcome of an analysis, risking the introduction of hidden researcher degrees of freedom and multiple unreported comparisons.

Here, we propose an alternative approach to identify a set of plausible combinations of brain regions for predicting behavioral data using a series of low‐dimensional multivariate models, avoiding mass voxelwise comparisons while still affording interpretable results.

## 1 Inference Versus Prediction

Generally, the goal of LSM analysis is to infer whether some regions of the brain are causally related to performance on a behavioral task; that is, whether damaging a particular region of the brain causes an especially meaningful change in task performance. Conventional LSM typically takes one of two forms. Mass‐univariate methods (Bates et al. 2003) test, at each voxel, the null hypothesis that the mean behavioral score does not differ between patients with and without damage. Typically, this is accomplished via a *t*‐test or Brunner‐Munzel test repeated independently across all voxels that are damaged in at least a pre‐determined percentage of individuals in the study cohort, with statistical power varying voxel‐to‐voxel as the sample size of individuals being studied changes (Kimberg et al. 2007). Multivariate methods such as SVR‐LSM (Zhang et al. 2014) instead enter all qualifying voxels simultaneously, assigning each a weight reflecting its contribution to a margin‐constrained prediction of behavior. Voxel weights surviving a permutation threshold constitute the best‐estimate network under this approach.

Both approaches require statistical thresholding and correction for multiple comparisons, which addresses the scenario in which no true effect exists yet false positives still arise by chance (Sperber et al. 2019). A second scenario is left unaddressed in both approaches: when a true effect exists for some tests in a set, neighboring regions can appear relevant merely through statistical co‐occurrence of damage—so‐called parasitic relationships or distortions—directly biasing interpretation. Both approaches are also vulnerable to lesion‐size confounds: a voxel may appear significant simply because it is frequently damaged in large lesions, acting as a proxy for diffuse damage rather than localized effect (DeMarco and Turkeltaub 2018; Sperber and Karnath 2017). Because larger lesions are expected to correlate with greater impairment independent of mechanism (Moore et al. 2024), the multiple recommended correction procedures (Mirman et al. 2018; Rorden et al. 2009) themselves become a source of variability in reported results.

In both general approaches described above, the end product of the statistical analysis pipeline is a best‐estimate map; a single solution is generated. An alternative approach involves model comparison. In this approach, multiple lesion‐behavior predictive pipelines are used, generating multiple solutions. These solutions can then be compared to evaluate which is best or whether there is no clear winner. Crucially, this model‐comparison framework shifts the unit of statistical inference from individual voxels to entire predictive maps. In this approach, we compare the results of different strategies for understanding the important features of the lesion data in predicting behavioral data.

## 2 Identifying Critical Networks for Lesion Symptom Mapping

Motivated by the opportunity to overcome the limitations of previous approaches, we introduce a predictive atlas‐based approach to lesion–symptom mapping that offers a complementary perspective on lesion–behavior relationships by emphasizing interpretable network‐level representations and explicit comparison among alternative models. We refer to the method as Critical Network Lesion Symptom Mapping (CN‐LSM). In short, the objective of CN‐LSM is to identify a set of prespecified regions from a neuroanatomical atlas that, when aggregated, best predict behavioral performance based on a proportional relationship with lesion damage within those regions. The approach is grounded in the neuroscientific concepts of cortical specialization, locally distributed processing, and functional connectivity and the statistical concepts of resampling and prediction (with less emphasis on statistical significance). The first assumption is that some areas of cortex are more important for behavior than others; that is, some areas incur more cost than others in terms of impairment on a given task when damaged. Supporting this assumption is the observation that different regions of the cortex have different cytoarchitectural and connectivity properties, and functional activation studies show preferential activation in different regions for different tasks. The second assumption is that processing is distributed over contiguous patches or volumes of tissue, so that damage within these regions and the corresponding behavioral impairment have a graded relationship (e.g., dose–response), rather than a binary all‐or‐none relationship. Neural network modeling studies and lesion studies support the idea that damaging different proportions of a distributed processing network leads to corresponding degrees of impairment (Rogers and McClelland 2014). The third assumption is that multiple cortical regions widely distributed across the cortex may work together as a single integrated processing network to accomplish a particular goal. Task‐based functional connectivity studies support this assertion, as well as the documented variability in locations of damage for the same symptom (e.g., word finding deficits) found in the lesion literature (Gleichgerrcht et al. 2017).

**FIGURE 1:**
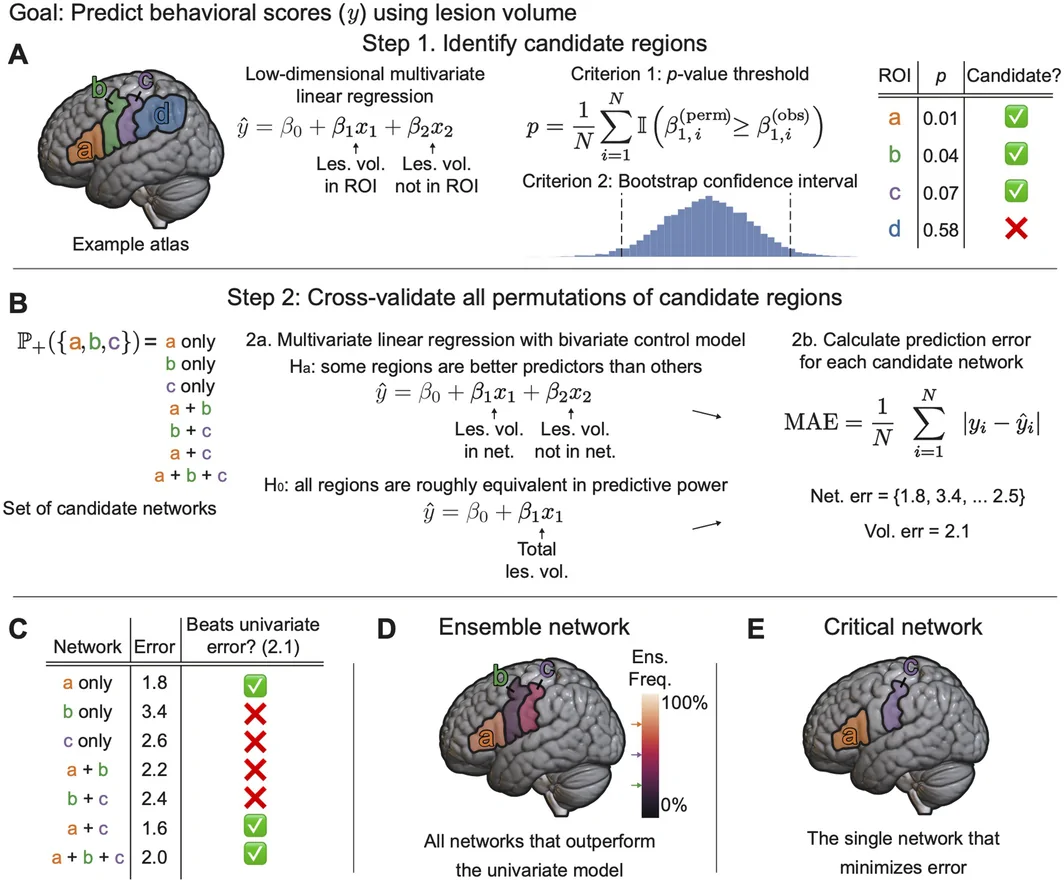
A schematic outlining the procedure for Critical Network Lesion‐Symptom Mapping (CN‐LSM). The goal of CN‐LSM is to predict behavioral scores from lesion data. The core assumption of the method is that some regions are more useful than others in predicting behavioral scores. (A) Each atlas region is evaluated through a permutation test and a bootstrap confidence interval which selects a set of candidate regions that meet specified *p*‐value and confidence thresholds. (B) Candidate networks are formed from all possible combinations of candidate regions. Prediction accuracy for each candidate network is evaluated via leave‐one‐out cross‐validation. For each network, a multivariate model is fit where behavioral scores are modeled using in‐network lesion volume (first coefficient) and out‐of‐network lesion volume (second coefficient). (C) The mean absolute error of each candidate network is tested for significance against the MAE of a simple linear model predicting behavioral scores using overall lesion volume. (D) The set of all network models that outperform the univariate lesion volume model forms an ensemble network model, from which individual region frequency and co‐occurrence within the ensemble can be calculated. (E) The single network that minimizes error relative to the univariate and ensemble prediction errors is declared the critical network.

Motivated by these assumptions, the CN‐LSM algorithm relies on the partial correlation between regional damage and a behavior. Figure 1 presents a schematic illustration of the algorithm. Specifically, a low‐dimensional multivariate linear model predicting behavior is fit with just two independent variables: (1) the amount of lesion within a region and (2) the amount of lesion outside that region. Formally,

$$y=\beta_{1}\sum_{r=1}^{R}x_{r}+\beta_{2}\sum_{k=1}^{K}x_{k}+\epsilon ,$$

where *y* is a continuous behavioral score, *x*_*r*_ is the lesion status of a voxel within the purported critical network, *x*_*k*_ is the lesion status of a voxel outside the purported critical network, *R* is the total number of critical voxels, *K* is the total number of noncritical voxels, and ϵ is an error term to be minimized. It is assumed that there are two possible costs associated with damage to a voxel, represented by the regression coefficients *β*_1_ and *β*_2_, depending on whether the voxel is critical (high cost) or noncritical (low cost) for the behavior, respectively. Notably, damage is not represented as relative proportions but rather as absolute frequencies. The key insight is that the entire lesion volume is not a confound; a portion of it is the signal of interest, and the goal is to separate this critically important tissue from less important tissue. We model out‐of‐region lesion (*β*_2_) precisely so that in‐region cost (*β*_1_) is estimated while holding diffuse damage constant; *β*_1_ is the parameter of interest because it isolates the localized signal. As a final check, the out‐of‐sample prediction error of the critical network model is compared to the error of the simple bivariate lesion volume regression model:

$$y=\beta_{1}\sum_{r=1}^{R}x_{r}+\epsilon .$$

The lesion volume confound problem is thus addressed through model comparison, positioning the simple bivariate lesion volume regression model as the standard to beat.

The CN‐LSM algorithm relies on a two‐step procedure to construct the critical network model. In the first step, a region or set of regions is identified as a potential contributor to the behavioral deficit of interest; we term these *candidate regions*. Candidate regions are identified using resampling methods, conceptually similar to a traditional mass univariate analysis of regions of interest. Specifically, for each region in the atlas (after excluding regions that do not meet a minimum threshold for number of participants with lesions), the multivariate linear regression model is fit to the data. A permutation test examines whether the *β*_1_ coefficient, corresponding to the behavioral cost of each of the potential candidate region's voxels being damaged, is larger than would be expected from randomly paired lesions and behavioral scores, yielding a *p*‐value. A bootstrap confidence interval examines whether the *β*_1_ coefficient remains negative (i.e., more damaged voxels imply lower behavioral scores) when a new sample of behavioral scores is generated from the original distribution with replacement. Both conditions must be met to be designated as a candidate region, improving the robustness of the statistical inference. We use the traditional thresholds for significance and robustness (i.e., permutation *p* < 0.05 and bootstrap 95th percentile < 0), although these can be relaxed to be more inclusive of smaller patient samples. It is important to remember that these thresholds are not the final determinants of inclusion in a resulting map; they merely serve as gatekeepers for the second step.

In the second step, if any candidate regions exist, all individual candidate regions and their possible combinations are evaluated for their predictive relationship with behavioral scores using cross‐validation. Cross‐validation is a flexible and robust method for comparing statistical models (Yarkoni and Westfall 2017). Specifically, we use leave‐one‐out cross‐validation (LOOCV) because it is efficient and yields unique results. With a large enough sample size, it is expected that the true causal associations with behavior will emerge as the most predictive model, while regions associated by proxy will not contribute additional predictive accuracy. The intention of this step is to remove parasitic associations that emerge due to the vascular nature of lesions (Sperber and Karnath 2017); however, evaluating all combinations of candidate regions grows exponentially (2^*n*^−1 models for n candidates), which could exceed practical compute limits. The first step mitigates this by reducing a whole‐brain analysis to a manageable candidate set; an optional cap (e.g., 15 candidates; 32,767 combinations) further bounds the search by ranking candidates on permutation *p*‐value.

The CN‐LSM algorithm additionally provides information about the set of networks that significantly outperform the bivariate lesion volume model (paired two‐tailed *t*‐test, *p* < 0.05) and are statistically indistinguishable from one another in their average prediction accuracies (paired two‐tailed *t*‐test, *p* > 0.05). We term this set of networks the *ensemble network*. While this approach technically yields multiple NHST comparisons due to the multiple candidate maps generated, the use of out‐of‐sample prediction mitigates some of the concern about “favorable” noise leading to false‐positive discoveries. More importantly, the goal is to identify a set of plausible networks using prediction accuracy as a proxy, rather than guarding against overinterpreting the significance of a single statistic that has been optimized through random sampling, which is the primary concern for multiple comparisons correction; the permutation tests in the first step serve that latter purpose. Analysis of the ensemble network allows researchers to identify frequently occurring regions and co‐occurrence of regions within high‐performing network models. Effectively, this enables zooming in on a portion of the results that has a high likelihood of being included in any plausible explanation, including the optimal recovery solution, which is typically unidentifiable in experimental paradigms where the ground truth is unknown.

## 3 Comparing LSM Approaches

We validated CN‐LSM by comparing it to the standard multivariate method, SVR‐LSM, using both ground‐truth simulation and real clinical data. Two considerations motivated this comparison. First, fewer researcher degrees of freedom can improve the consistency of findings on their own, and providing an alternative perspective on the same data allows results to be combined across methods to counterbalance each one's limitations. Second, in a predictive paradigm, model flexibility is not necessarily advantageous: the more the model space is constrained to reflect the data‐generating process (i.e., theory), the more likely an estimated model is to generalize to unseen data. The high‐dimensional flexibility of SVR‐LSM—along with components unrelated to localization (e.g., tolerance margins, nonlinear transformations, and the use of positive and negative associations)—may improve prediction without capturing true functional localization. For these reasons, our primary focus is the recovery of ground truth, with prediction accuracy considered secondarily. The researcher degrees of freedom for each method are listed in Table 1; both require decisions about brain parcellation and the statistical certainty required for inference, but SVR‐LSM requires several additional decisions regarding multiple comparisons, noncontiguous results, lesion‐volume correction, indeterminacy from having more predictors than observations, nonlinear transformations, and, optionally, interpretation of the results with an atlas.

**TABLE 1:** Researcher degrees of freedom.

SVR‐LSM	CN‐LSM
Voxel size	Atlas
2 Number of lesions per voxel	2 Number of lesions per region
3 Number of permutations	3 Number of permutations
4 Permutation percentile	4 Permutation percentile
5 FDR q threshold	5 (optional) Maximum number of candidates
6 Cluster size	
7 Volume correction method	
8 C	
9 γ	
10 (optional) Atlas for interpretation	

## 4 Methods

### 4.1 Simulation Experiment

Ground‐truth simulation was used to compare CN‐LSM with SVR‐LSM. The ground truth is usually unknown in clinical studies with real behavioral data. However, in a ground‐truth simulation, one can use real neuroimaging data (thus incorporating typical lesion distributions) together with simulated relationships between behavioral scores and their neural correlates. There have been many such studies, with variations in the simulated ground‐truths based on their goals (Inoue et al. 2014; Ivanova et al. 2021; Mah et al. 2014; Mirman et al. 2018; Pustina et al. 2018; Sperber 2020; Sperber et al. 2019; Sperber and Karnath 2017; Sperber and Umarova 2024; Zavaglia et al. 2024; Zhang et al. 2014).

#### 4.1.1 Participants

**FIGURE 2:**
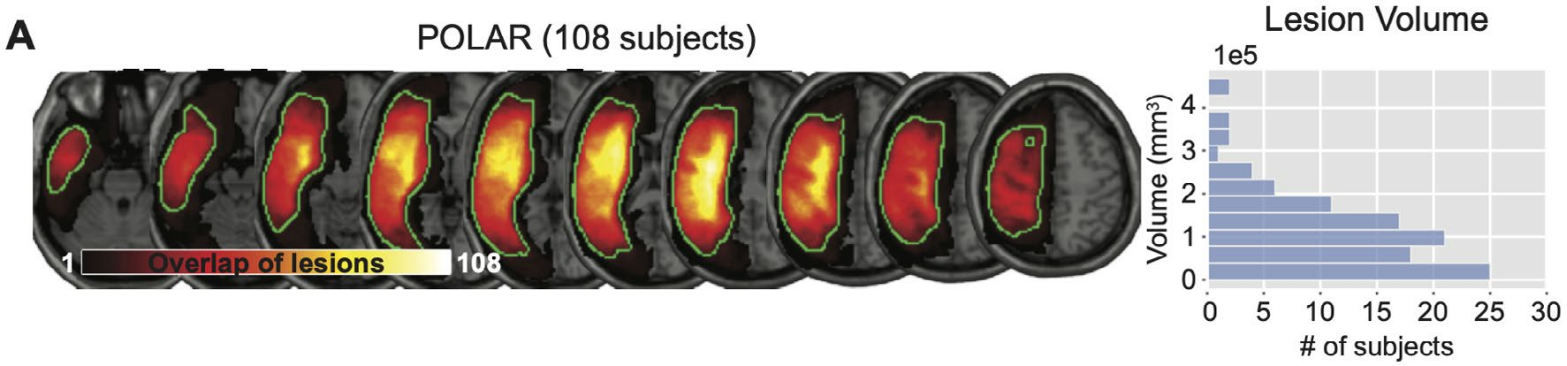
Lesion coverage maps for the POLAR cohort, generated by the DeMarco and Turkeltaub (2018) toolbox. The green outline encompasses voxels with 10 or more overlapping lesions. One participant was excluded from the cohort maps due to not having any voxels in the minimum lesion cutoff mask. This individual's data were included in the CN‐LSM analyses but not in the SVR‐LSM analyses. To the right is a histogram of lesion mask volume.

Lesion data from 109 participants with a single, chronic (> 12 mos.) left hemisphere stroke were included from the Predicting Outcomes of Language Rehabilitation (POLAR) clinical trial (Kristinsson et al. 2023). Lesion overlap maps and distributions of lesion mask volumes for 108 participants are shown in Figure 2 (one participant was excluded due to the lesion not overlapping with the voxels that exceeded the threshold of minimum lesions).

#### 4.1.2 Target Brain Regions

**FIGURE 3:**
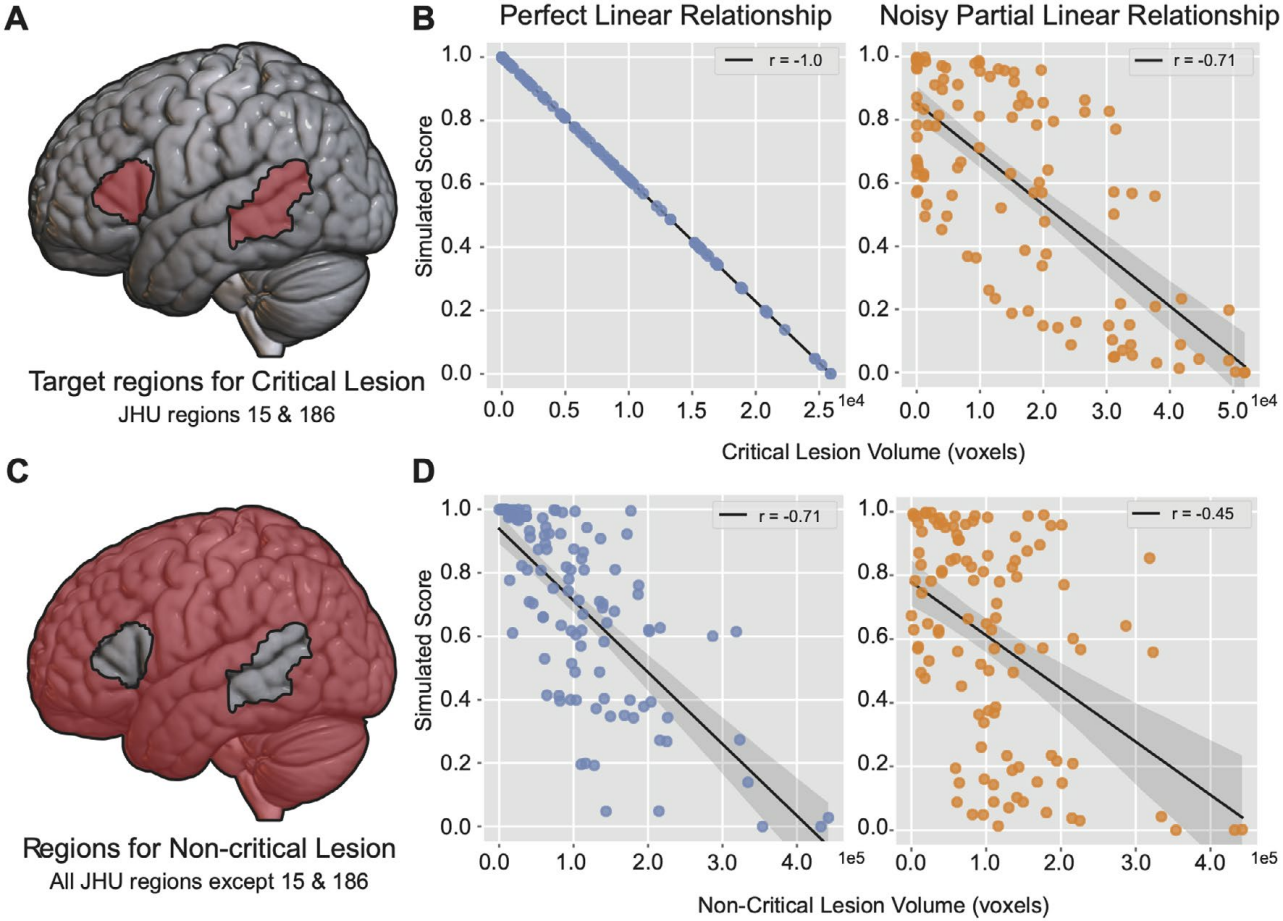
Target voxels (A) and nontarget voxels (C) for the simulated data experiment are displayed at the left of the figure (top and bottom). Regions 15 (Inferior Frontal Gyrus Pars Triangularis) and 186 (Posterior Middle Temporal Gyrus) from the JHU atlas were selected as the target voxels. Scatterplots showing relationships between damage to target voxels and the simulated behavioral scores with perfect or noisy relationships to target voxels are displayed on top (B). Scatterplots showing relationships between damage to nontarget voxels and the simulated behavioral scores with perfect or noisy relationships to target voxels are displayed on the bottom (D).

We chose two regions (15—Inferior Frontal Gyrus Pars Triangularis, 186—Posterior Middle Temporal Gyrus) from the Johns Hopkins University atlas distributed with the NiiStat software (https://www.nitrc.org/projects/niistat/) to serve as neuroanatomical targets for the simulations to recover. These regions are frequently implicated in studies of language and are often hypothesized to function as a distributed network supporting language processing. The combined target region included 25,904 1 mm^3^ voxels (Figure 3A).

#### 4.1.3 Simulated Behavioral Scores

Scores were simulated with two possible relations to the frequency of damaged voxels in the target region.

1. A perfect linear relationship (Figure 3B,D, left column):
$$y_{i}=-x_{i},$$
  where $y_{i}$ is the simulated score for participant *i*, and $x_{i}$ is the number of damaged voxels in the target regions for participant *i*. Scores $y_{i}$ were translated and scaled to the [0,1] interval by subtracting the minimum score value and dividing by the maximum score value.
2. A noisy, partial linear relationship (Figure 3B,D, right column):
$$y_{i}=-x_{i}+\epsilon ,$$
  where $y_{i}$ and $x_{i}$ are as before, and ϵ is heteroscedastic measurement noise. This relationship was achieved through a series of transformations. Initially, the frequencies of damaged critical voxels were translated and scaled to the range [0, 1] (i.e., subtracting the minimum frequency and dividing by the maximum frequency), then multiplied by 0.95 to remove ceiling effects. Then, score values were converted to the probit scale using the inverse normal cumulative distribution function. A random draw from a uniform distribution centered on zero was added to each probit value. To approximate realistic levels of noise, the width of the uniform distribution interval was determined through informal comparison of the resulting proportion of variance explained (*r* ^2^) by the optimal CN‐LSM model in simulated data to the proportion of variance explained in real data (e.g., using the AALCAT atlas, *r* ^2^ = 0.5127 for WAB fluency, and *r* ^2^ = 0.3789 for WAB comprehension). Manually adjusting the uniform distribution interval width to yield comparable results in simulated scores across multiple random samples indicated an appropriate interval width of three probits. Then, values were converted back to the [0, 1] scale using the normal cumulative distribution function. The effect of these transformations was to include heteroscedastic noise, as typically seen in behavioral measurement on a closed interval (i.e., a test score range), with more noise (i.e., greater test–retest variability) in the middle of the scale and less near the extremes.

#### 4.1.4 Statistical Mapping Methods

In independent runs of CN‐LSM, we used the AICHA atlas (Joliot et al. 2015) and the AALCAT atlas (Yourganov et al. 2015), both distributed with NiiStat. The AICHA atlas is a gray matter atlas that does not include specific white matter tracts (effectively relegating these to noncritical status), although white matter underlying the cortex is included in most parcels. The AALCAT atlas parcellates both gray matter and white matter tracts. Notably, we did not use the JHU atlas from which the target voxels were selected, reflecting the typical situation where the true parcellation of the function under investigation is unknown. Regions where at least 10 participants had damage were included. Resampling for permutation and bootstrap tests included 10^4^ iterations, with thresholds of *p* < 0.05 and 95th percentile < 0 for permutations and bootstrap tests, respectively. A maximum candidate region limit of 15 candidates was implemented.

For SVR‐LSM, we used the implementation provided by DeMarco and Turkeltaub (2018). The DeMarco and Turkeltaub (2018) implementation relies on permutation testing for inference. We used default parameter settings, except for the number of permutations to reduce analysis durations. The lesion masks were first down sampled by a factor of 2.5 along each dimension, as recommended by A. DeMarco (personal communication, May 21, 2025), reducing the number of imaging features (i.e., voxels) in the model and the time required to fit it. Voxels with at least 10 lesions were included. A *C* parameter of 30 and a *γ* parameter of 5 were used. To obtain timely results, 2000 samples were included for permutation tests with a voxelwise *p‐*value threshold of 0.005 and a clusterwise *p*‐value threshold of 0.05. Lesion volume was regressed out of the behavior and the lesion data. This implementation uses the average of 10 replications of five‐fold cross‐validation to estimate prediction error; it was assumed these values were approximately comparable to leave‐one‐out estimates.

#### 4.1.5 Outcome Measures

##### 4.1.5.1 Voxel‐Level Comparison

Three measures of overlap between the voxels identified through lesion‐symptom mapping and the target voxels are reported. The *positive predictive value* (PPV) is reported as the proportion of identified voxels that overlap target voxels (i.e., low values indicate many false positive voxels). The *target coverage* (TC) is reported as the proportion of target voxels that overlap with identified voxels (i.e., low values indicate many false negative voxels). The *Dice index* (F1) is reported as two times the number of intersecting voxels, divided by the sum of the number of identified voxels and the number of target voxels. This is the primary statistic used to compare the methods for successful topographical inference, as it balances both PPV and TC, which tend to trade off. For example, identifying all voxels as critical would have high TC and low PPV, while identifying a single target voxel would have high PPV and low TC; neither of these options would yield a high F1, which is maximized when there is perfect overlap between target and identified voxels. Cross‐validation prediction accuracy (i.e., mean absolute error) for the simulated scores is also reported.

For CN‐LSM, these statistics were examined with respect to the solution that yields optimal prediction accuracy (i.e., the critical network) as well as the solution within the ensemble that yields optimal target voxel recovery (noting that this is only possible in a simulation paradigm where the true voxels are known). To account for the effects of simulated measurement noise, each LSM procedure was repeated 10 times on separate behavioral datasets generated from the same noisy lesion‐behavior relationship.

##### 4.1.5.2 ROI‐Level Comparison and Validation of the Ensemble Inclusion Rate

Because CN‐LSM and SVR‐LSM produce results at different native resolutions (atlas regions vs. voxels), we additionally evaluated both methods on a common ROI footing and assessed the criterion validity of the CN‐LSM ensemble inclusion rate against the established SVR‐LSM indicator, the proportion of significant voxels within a region. For each simulated dataset and atlas, we classified atlas regions into three categories defined by overlap with the target voxels: *Strong Hits*, the region set that maximizes F1 overlap with the targets (the ROI‐level “correct answer”); *Weak Hits*, regions with some target overlap but insufficient to enter the F1‐maximizing set; and *False Positives*, identified regions with no target overlap. For each indicator (i.e., the ensemble inclusion rate or the proportion of significant voxels) we compared the mean value for Strong Hits versus the combined Weak Hits + False Positives using unpaired, two‐sample *t*‐tests, and computed ROC curves with the area under the curve (AUC), reporting the true‐ and false‐positive rates at the optimal threshold. SVR‐LSM regions were overlaid on the AICHA and AALCAT atlases for this comparison. Full per‐dataset frequency counts and proportions are provided in Supporting Informations S1.

### 4.2 Real Data Experiment

A potential weakness of the simulation approach is that the typically simplified assumptions may not match the important complexities of real clinical data. We thus sought to validate CN‐LSM using real data as well. In their seminal paper presenting and validating the VLSM method, Bates et al. (2003) examined fluency and comprehension scores from the Western Aphasia Battery (Kertesz 1982). These scores were examined because they were known to dissociate behaviorally in aphasia, and previous investigations using lesion overlap methods provided strong evidence that these functions localize to distinct frontal areas and temporal areas, respectively. For these reasons, the same scores (from different participants) were examined here.

#### 4.2.1 Participants

The same 109 participants from the simulation experiment were included, this time using real behavioral scores. They were evaluated behaviorally by a team of speech‐language pathologists. See Spell et al. (2020) for a thorough description of the complete dataset. Clinical and demographic information is provided in Table 2.

**TABLE 2:** Participant demographic and clinical descriptive statistics.

Sample	Total
*N*	109
Sex	Frequency (%)
Male	64 (59%)
Female	45 (41%)
Handedness	
Ambidextrous	1 (1%)
Left	10 (9%)
Right	98 (90%)
Race	
Asian	1 (1%)
Black	25 (23%)
White	83 (76%)
WAB aphasia type	
Anomia	27 (25%)
Broca's	45 (41%)
Conduction	13 (12%)
Global	5 (5%)
Wernicke's	6 (6%)
Transcortical motor	1 (1%)
No aphasia	12 (11%)

Other demographic variables	Mean	Standard deviation	Minimum	Maximum
Time post stroke (months)	53.38	53.55	10.00	241.00
Age (years)	61.01	10.99	29.00	80.00
Education (years)	15.55	2.32	12.00	20.00
WAB Aphasia Quotient	63.34	24.88	14.50	100.00
NIH Stroke Scale	5.89	3.97	0.00	18.00
WAB Fluency	5.42	2.88	1.00	10.00
WAB Auditory Verbal Comprehension	7.92	1.83	2.95	10.00

#### 4.2.2 Behavioral Assessment

We separately examined Western Aphasia Battery‐Revised (Kertesz 2007) scores for Fluency and for Comprehension. Fluency was rated on an ordinal 10‐point scale based on clinical judgment after observing narrative speech elicitation tasks, including picture description and open‐ended questions (e.g., “What brings you here today?”). Comprehension was measured as a composite score of several tasks, including pointing to named objects and pictures, answering yes/no questions, and following sequential commands. Notably, while intending to measure auditory comprehension, two tasks (i.e., pointing to named objects and following commands) also strongly relied on visual processing.

#### 4.2.3 Lesion‐Symptom Mapping

We applied CN‐LSM with the AALCAT, AICHA, and JHU atlases, using the same parameters as in the simulation experiment. We also applied the (DeMarco and Turkeltaub 2018) implementation of SVR‐LSM using the same parameters as in the simulation experiment. Based on previous reports (Bates et al. 2003; DeMarco and Turkeltaub 2018), fluency scores were expected to localize to anterior dorsal stream areas in the frontal lobe, particularly near the motor cortex controlling the speech effectors; comprehension scores were expected to localize to posterior ventral stream areas in the temporal lobe, particularly near the superior and middle temporal gyri.

## 5 Results

### 5.1 Simulation Experiment

#### 5.1.1 Voxel‐Level Comparison

The overlap statistics (PPV, TC, and F1, respectively) are presented in Tables 3, 4, 5, and the prediction accuracy statistics are presented in Table 6. Regarding the primary overlap statistic (F1), in the No Noise condition, the optimal prediction model from CN‐LSM using AALCAT (F1 = 0.4704) and AICHA (F1 = 0.3988) both outperformed SVR‐LSM (F1 = 0.3074). This pattern was mirrored in the prediction accuracies as well: CN‐LSM using AALCAT (MAE = 0.0487) and AICHA (MAE = 0.0789) yielded smaller average prediction errors than SVR‐LSM (MAE = 0.082). Examining the PPV and TC statistics revealed that, in the No Noise condition, SVR‐LSM was anti‐conservative, yielding relatively high TC (0.7598) but low PPV (0.1927). Compared to SVR‐LSM, CN‐LSM using AALCAT had slightly larger TC (0.7859) and substantially larger PPV (0.3356), while CN‐LSM using AICHA struck a balance between these statistics (TC = 0.3611, PPV = 0.4453).

**TABLE 3:** Positive predictive value (PPV).

	PPV				
	AICHA	AICHA_Ensemble	AALCAT	AALCAT_Ensemble	SVR‐DT
No noise	0.4453	0.5916	0.3356	0.3733	0.1927
Noise_1	0.359	0.5395	0.2557	0.3366	0.2771
Noise_2	0	0	0.249	0.2776	0.3188
Noise_3	0.4445	0.4922	0.249	0.3733	0.2753
Noise_4	0.3747	0.5395	0.2472	0.3366	0.2781
Noise_5	0.4324	0.5395	0.103	0.3366	0.2997
Noise_6	0.126	0.3046	0.0274	0.1508	0.1713
Noise_7	0.0009	0.3779	0.249	0.3733	0.2895
Noise_8	0.2388	0.5419	0.2593	0.3733	0.2589
Noise_9	0.3745	0.4648	0.2579	0.3716	0.3825
Noise_10	0.4205	0.385	0.1822	0.3366	0.3024
Noise_mean	0.27713	0.41849	0.20797	0.32663	0.28536
Noise_min	0	0	0.0274	0.1508	0.1713
Noise_max	0.4445	0.5419	0.2593	0.3733	0.3825

*Note:* AICHA and AALCAT are atlases used with CN‐LSM, and the optimal prediction model is reported. The optimal recovery model in terms of F1 is reported for the ensembles derived from these analyses.

**TABLE 4:** Target coverage (TC). AICHA and AALCAT are atlases used with CN‐LSM, and the optimal prediction model is reported.

	TC				
	AICHA	AICHA_Ensemble	AALCAT	AALCAT_Ensemble	SVR‐DT
No noise	0.3611	0.3362	0.7859	0.7859	0.7598
Noise_1	0.1068	0.428	0.4917	0.4917	0.5416
Noise_2	0	0	0.4052	0.8047	0.4701
Noise_3	0.5077	0.5864	0.8293	0.7859	0.6657
Noise_4	0.2504	0.428	0.4922	0.4917	0.5559
Noise_5	0.3309	0.428	0.0545	0.4917	0.5738
Noise_6	0.1587	0.3244	0.0206	0.3577	0.099
Noise_7	0.0002	0.3527	0.4052	0.7859	0.4604
Noise_8	0.2486	0.4729	0.8086	0.7859	0.4597
Noise_9	0.3885	0.6212	0.7534	0.7314	0.5647
Noise_10	0.1313	0.399	0.4371	0.4917	0.5845
Noise_mean	0.21231	0.40406	0.46978	0.62183	0.49754
Noise_min	0	0	0.0206	0.3577	0.099
Noise_max	0.5077	0.6212	0.8293	0.8047	0.6657

*Note:* The optimal recovery model in terms of F1 is reported for the ensembles derived from these analyses.

**TABLE 5:** Dice overlap (F1). AICHA and AALCAT are atlases used with CN‐LSM, and the optimal prediction model is reported.

	F1				
	AICHA	AICHA_Ensemble	AALCAT	AALCAT_Ensemble	SVR‐DT
No noise	0.3988	0.4288	0.4704	0.5062	0.3074
Noise_1	0.1646	0.4774	0.3364	0.3996	0.3666
Noise_2	0	0	0.3084	0.4128	0.3799
Noise_3	0.474	0.5352	0.383	0.5062	0.3895
Noise_4	0.3002	0.4774	0.3291	0.3996	0.3708
Noise_5	0.3749	0.4774	0.0713	0.3996	0.3937
Noise_6	0.1405	0.3142	0.0235	0.2121	0.1255
Noise_7	0.0003	0.3649	0.3084	0.5062	0.3555
Noise_8	0.2436	0.5051	0.3927	0.5062	0.3313
Noise_9	0.3814	0.5318	0.3843	0.4928	0.4561
Noise_10	0.2001	0.3919	0.2572	0.3996	0.3986
Noise_mean	0.22796	0.40753	0.27943	0.42347	0.35675
Noise_min	0	0	0.0235	0.2121	0.1255
Noise_max	0.474	0.5352	0.3927	0.5062	0.4561

*Note:* The optimal recovery model in terms of F1 is reported for the ensembles derived from these analyses.

**TABLE 6:** Prediction mean absolute error (MAE).

	MAE				
	AICHA	AICHA_Ensemble	AALCAT	AALCAT_Ensemble	SVR‐DT
No noise	0.0789	0.0889	0.0487	0.051	0.082
Noise_1	0.1854	0.1923	0.192	0.1927	0.1414
Noise_2	0	0	0.1778	0.1858	0.1266
Noise_3	0.1437	0.1565	0.1519	0.1628	0.1039
Noise_4	0.1799	0.1829	0.1838	0.1847	0.1337
Noise_5	0.2164	0.22	0.2188	0.2229	0.1572
Noise_6	0.1795	0.1838	0.1832	0.1844	0.1275
Noise_7	0.2013	0.2126	0.1832	0.204	0.1437
Noise_8	0.1832	0.1902	0.1908	0.1943	0.1399
Noise_9	0.1837	0.1933	0.1859	0.197	0.1492
Noise_10	0.1723	0.1792	0.1771	0.1826	0.1234
Noise_mean	0.16454	0.17108	0.18445	0.19112	0.13465
Noise_min	0	0	0.1519	0.1628	0.1039
Noise_max	0.2164	0.22	0.2188	0.2229	0.1572

*Note:* AICHA and AALCAT are atlases used with CN‐LSM, and the optimal prediction model is reported. The optimal recovery model in terms of F1 is reported for the ensembles derived from these analyses.

In the Realistic Noise condition, on average, SVR‐LSM (mean F1 = 0.3568) outperformed the optimal prediction model from CN‐LSM using AALCAT (mean F1 = 0.2794) and using AICHA (mean F1 = 0.228). Notably, when using AICHA for CN‐LSM, one behavioral data set did not yield any candidates that outperformed the simple lesion volume control model, resulting in no overlap statistics and zeros (i.e., demonstrating the conservative nature of the approach). However, within the ensemble of CN‐LSM models, at least one model consistently outperformed SVR‐LSM in ground truth recovery, using AALCAT (10/10 datasets, mean max. F1 = 0.4235) or AICHA (8/10 datasets, mean max. F1 = 0.4075). The AALCAT ensemble included a network with the maximum possible F1 (0.5062) in 3/10 datasets, and the AICHA ensemble included a network that approached the maximum possible F1 (0.5382) in 2/10 datasets; F1 for SVR‐LSM results (maximum F1 = 0.4561) consistently fell below the maximum possible F1 when using an atlas (0.5602 or 0.5382). Of particular interest, these optimal recovery models were never the optimal prediction models. Despite the improved ground‐truth recovery, CN‐LSM yielded greater prediction errors than SVR‐LSM (mean MAE = 0.1347) when using AALCAT (mean MAE = 0.1911) or AICHA (mean MAE = 0.1711).

#### 5.1.2 ROI‐Level Comparison and Validation of the Ensemble Inclusion Rate

The CN‐LSM indicator (ensemble inclusion rate) significantly distinguished Strong Hits from Weak Hits + False Positives using both the AICHA atlas (*M* = 0.536 vs. 0.471; *t* = 2.17, *p* = 0.032; *AUC* = 0.669) and the AALCAT atlas (*M* = 0.634 vs. 0.492; *t* = 3.49, *p* < 0.001; *AUC* = 0.747). The SVR‐LSM indicator (proportion of significant voxels) showed the same pattern with sharper separation (AICHA: *M* = 0.602 vs. 0.168; *t* = 12.56, *p* < 0.001; *AUC* = 0.846; AALCAT: *M* = 0.533 vs. 0.090; *t* = 13.96, *p* < 0.001; *AUC* = 0.944). Although the SVR‐LSM indicator was more discriminating, its statistical advantage was partially because overlaying the SVR‐LSM voxel‐level results on any atlas without thresholding yielded dramatically more false positive regions to begin with—by a factor of 20 for AICHA and a factor of 7 for AALCAT—than the optimal CN‐LSM network, which guards against false positives at the region‐level with permutation tests. In classification terms, the negative class had many more instances to contribute to the classification statistics. Still, when the optimal threshold for the proportion of significant voxels was applied to SVR‐LSM, it did achieve a higher true positive rate than the ensemble inclusion rate for CN‐LSM, with all methods achieving a comparable false positive rate between 3% and 4%.

**FIGURE 4:**
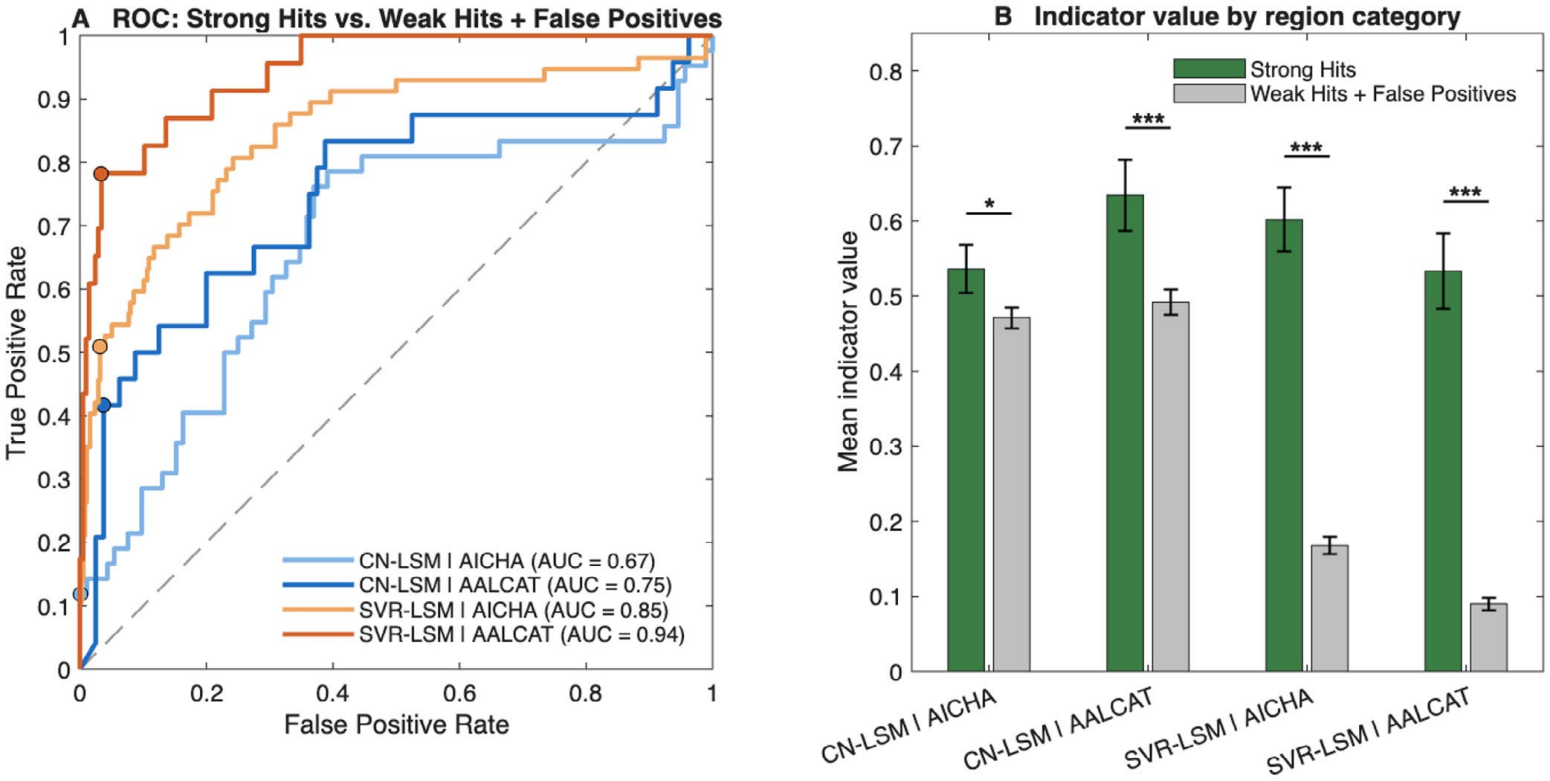
Atlas regions were classified by overlap with the ground‐truth targets as Strong Hits (the F1‐maximizing region set), Weak Hits (partial overlap), or False Positives (no overlap); SVR‐LSM results were overlaid on the AICHA and AALCAT atlases for this comparison. (A) ROC curves for each indicator's ability to distinguish Strong Hits from Weak Hits + False Positives—the CN‐LSM ensemble inclusion rate (blues) and the SVR‐LSM proportion of significant voxels (oranges)—with AUCs in the legend and filled circles marking each optimal operating point. (B) Mean indicator value (± SE) for Strong Hits versus Weak Hits + False Positives. Both indicators significantly separated true from spurious regions (*p < 0.05, ***p < 0.001).

Although the optimal threshold for the ensemble inclusion rate using AICHA was 92% (TPR = 0.119, FPR = 0), the lower threshold identified for AALCAT (70%) could be used consistently across both atlases (AICHA: TPR = 0.143, FPR = 0.033; AALCAT: TPR = 0.417, FPR = 0.038), whereas the optimal coverage threshold for the SVR‐LSM indicator differed substantially by atlas (75% for AICHA: TPR = 0.509, FPR = 0.032; 40% for AALCAT: TPR = 0.783, FPR = 0.034). Unlike the SVR‐LSM indicator, calculation of the CN‐LSM indicator does not depend directly on the shape and size of an atlas region. Thus, the CN‐LSM ensemble inclusion rate is a valid ROI‐level indicator in the same sense as the proportion of significant voxels—significantly separating true from spurious regions—while deriving from an entirely different set of assumptions and offering a threshold that transfers across atlases (Figure 4).

Across the 10 datasets, when using AALCAT, there were six regions that emerged with ensemble inclusion rates > 70%: inferior frontal gyrus (IFG) pars triangularis, IFG pars opercularis, middle temporal gyrus (MTG), arcuate fasciculus, posterior segment of the superior longitudinal fasciculus (SLF), and superior temporal pole. All of these regions overlapped with the ground truth voxels, except the superior temporal pole, which was directly adjacent (ventral) to the target region in the frontal lobe. When using AICHA, there were five regions that emerged with ensemble inclusion rates > 70%: IFG pars triangularis, superior temporal sulcus (two separate regions), MTG, and the middle temporal pole. Again, all of these regions overlapped with the ground truth voxels, except the middle temporal pole, which was directly adjacent (anterior) to the target region in the temporal lobe.

### 5.2 Real Data Experiment

**FIGURE 5:**
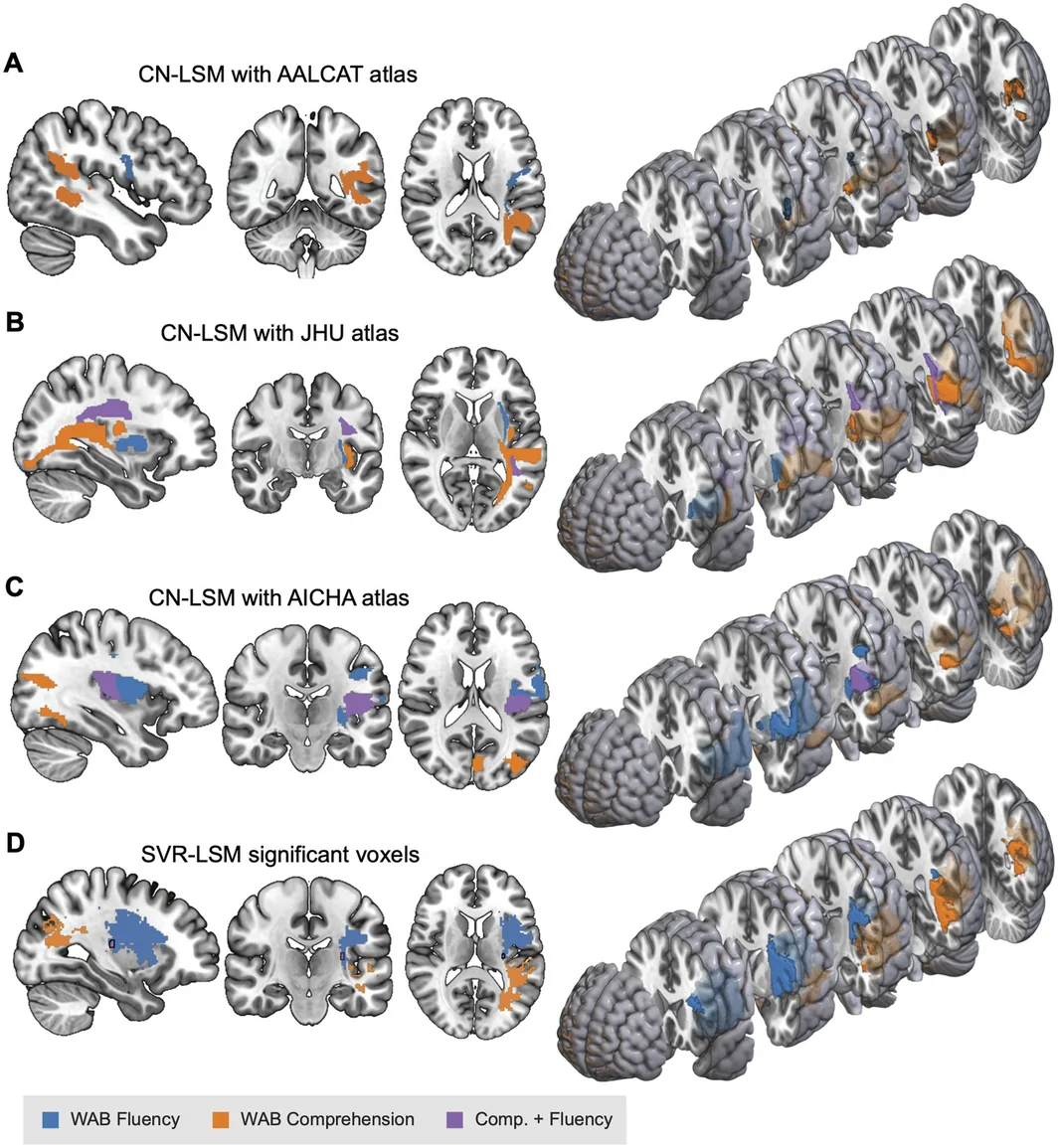
CN‐LSM results for optimal prediction of WAB Fluency (blue) and WAB Comprehension (orange) and their overlap (purple) using the (A) AALCAT atlas, (B) JHU atlas, and (C) AICHA atlas, as well as the (D) SVR‐LSM significant voxels.

First, we consider the models that yielded the best predictions of fluency and comprehension scores. CN‐LSM with AALCAT, AICHA, and JHU yielded significantly better predictions than the simple lesion volume control model, with the AALCAT and JHU atlases (which both include white matter tracts) providing comparably superior predictions to the AICHA atlas. Using AALCAT, comprehension localized to optic radiations and posterior segment of the superior longitudinal fasciculus (SLF) (MAE = 1.098), while fluency localized to the long segment of the SLF (MAE = 1.664) (Figure 5A). Using JHU, comprehension localized to superior and inferior occipital gyri, retrolenticular part of internal capsule, posterior thalamic radiation, SLF, posterior insula, and posterior superior temporal gyrus (STG) (MAE = 1.090), while fluency localized to external capsule and SLF (MAE = 1.675) (Figure 5B). Using AICHA, comprehension localized to superior temporal sulcus, inferior temporal gyrus, occipital middle gyrus, fusiform gyrus, intraoccipital sulcus, parietooccipital sulcus, calcarine gyrus, and posterior insula (MAE = 1.131), while fluency localized to Rolandic sulcus, posterior insula, Rolandic operculum, and putamen (MAE = 1.829) (Figure 5C).

To interpret the SVR‐LSM results, the significant voxels were partitioned using the AALCAT atlas. Nineteen regions were implicated for comprehension, including Rolandic operculum, insula, occipital lobe (superior and middle), angular gyrus, Heschl's gyrus, temporal gyri (superior, middle, and inferior), SLF (posterior and long segments), arcuate fasciculus, corpus callosum, cortico‐ponto‐cerebellum tract, cortico‐spinal tract, inferior longitudinal fasciculus, inferior occipito‐frontal fasciculus, internal capsule, and optic radiations (MAE = 0.690). Unlike CN‐LSM, SVR‐LSM assigns various effect sizes to different voxels; however, in practice there was very little variation among estimated effect sizes, with only 10 unique *Z*‐scores [−3.29, −2.58] assigned to 23,704 different voxels. All implicated regions included peak values. For fluency, 20 regions were implicated, including precentral gyrus, IFG (opercularis and triangularis), Rolandic operculum, insula, postcentral gyrus, inferior parietal lobe, supramarginal gyrus, putamen, Heschl's gyrus, STG, SLF (anterior and long segments), arcuate fasciculus, cortico‐ponto‐cerebellum tract, cortico‐spinal tract, inferior occipito‐frontal fasciculus, internal capsule, optic radiations, and uncinate fasciculus (MAE = 1.327) (Figure 5D). Like the comprehension measure, the same 10 unique *Z*‐scores were assigned to 29,944 voxels, with all identified regions containing peak values. While many of the same atlas regions were implicated for the two behavioral scores using SVR‐LSM, only 112 out of 53,536 total implicated voxels overlapped (0.2%); these were contained within the SLF (anterior segment), arcuate fasciculus, cortico‐ponto‐cerebellum tract, cortico‐spinal tract, and internal capsule.

**FIGURE 6:**
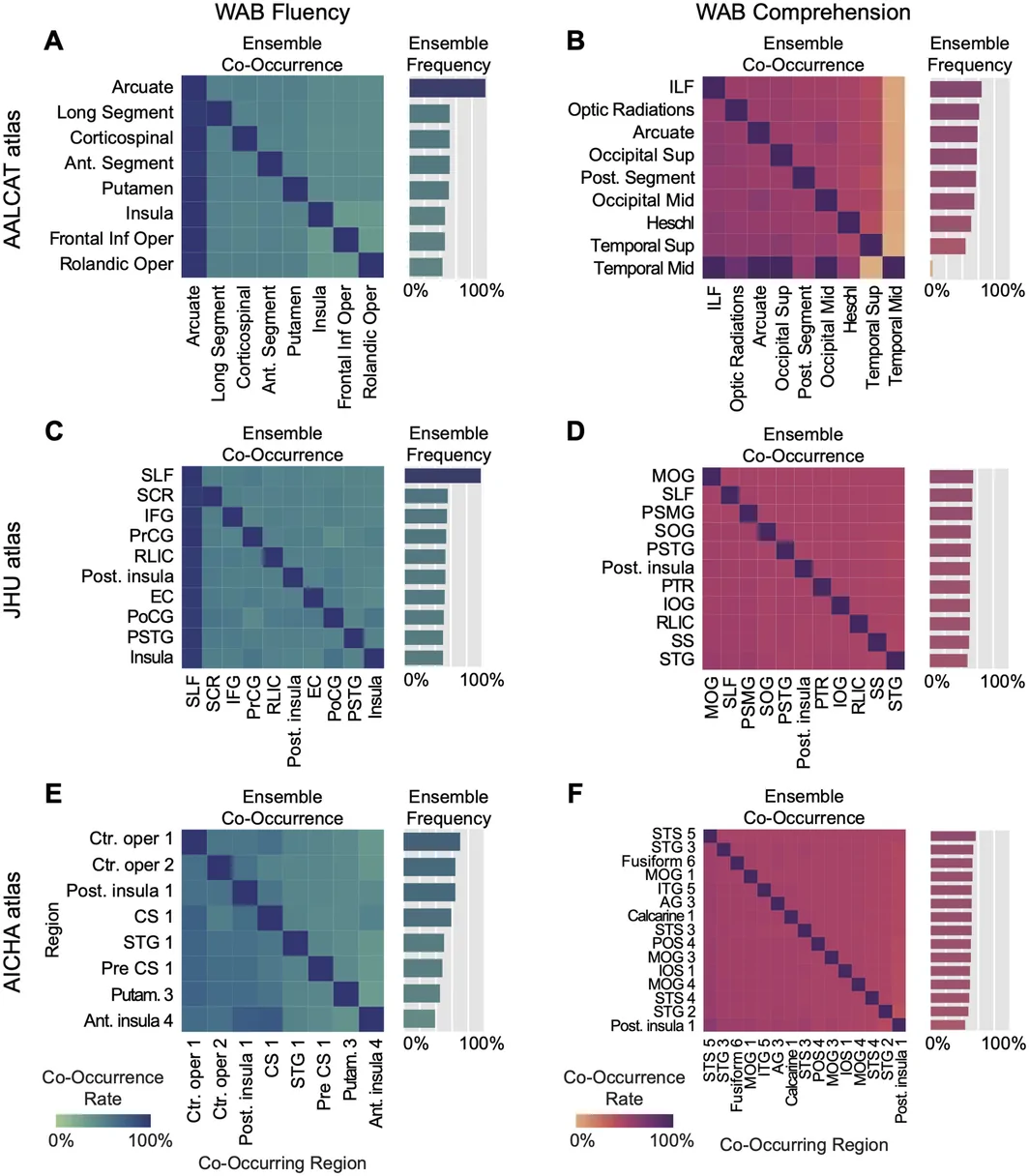
Ensemble networks for WAB fluency (left column, panels A, C, and E) and Comprehension (right column, panels B, D, and F) using the AALCAT atlas (top row, panels A and B), JHU atlas (middle row, panels C and D), and AICHA atlas (bottom row, panels E and F). Ensemble co‐occurrence matrices display the proportion of models that included a region on the *y*‐axis (rows) that also included a region on the *x*‐axis (columns). Ensemble frequency bar graphs display the proportion of all models that included a given region. Abbreviated region names refer to atlas labels.

Noting that in the simulations, the optimal prediction models were not optimal at recovering target voxels, we turn next to the analysis of the ensemble of network models. The ensemble network results using the AALCAT atlas are shown in Figure 6 (top row). Using the AALCAT atlas for fluency (Figure 6A), beyond the single white matter tract included in the optimal prediction model, an additional seven regions emerged: IFG pars opercularis, Rolandic operculum, insula, putamen, anterior segment of the SLF, arcuate fasciculus, and corticospinal tract. The arcuate fasciculus had an ensemble inclusion rate of 97%, which is notable given that this region was not included in the optimal prediction model. Using the AALCAT atlas for comprehension (Figure 6B), beyond the two white matter regions included in the optimal prediction model, an additional seven regions emerged: superior and middle occipital gyri, Heschl's gyrus, superior and middle temporal gyri, arcuate fasciculus, and inferior longitudinal fasciculus (ILF). The ILF had the maximum ensemble inclusion rate (64%).

Using the JHU atlas for fluency (6C), beyond the two white matter tracts included in the optimal prediction model, an additional eight regions emerged: IFG pars opercularis, postcentral gyrus, precentral gyrus, insula, superior corona radiata, retrolenticular part of the internal capsule, posterior insula, and posterior STG. The SLF had an ensemble inclusion rate of 98%. Using the JHU atlas for comprehension (6D), beyond the seven regions included in the optimal prediction model, an additional five regions emerged: STG, middle occipital gyrus, sagittal stratum, and posterior MTG. The middle occipital gyrus had the maximum ensemble inclusion rate (55%).

Using the AICHA atlas for fluency (6E), beyond the five regions included in the optimal prediction model, an additional three regions emerged: precentral gyrus, anterior insula, and STG. The Rolandic operculum had an ensemble inclusion rate of 70%. Using the AICHA atlas for comprehension (6F), beyond the nine regions included in the optimal prediction model, an additional six regions emerged: two regions of STG, two regions of superior temporal sulcus, angular gyrus, and middle occipital gyrus. The superior temporal sulcus (region 5) had the maximum ensemble inclusion rate (57%).

## 6 Discussion

### 6.1 Summary

The present study introduced CN‐LSM as a technique for modeling lesion‐behavior relationships. We do not present CN‐LSM as a reinvention of the wheel or the best way to do LSM; we view it as a complementary method to established techniques that can help converge on results due to its relatively small set of assumptions and its alternative perspective on statistical inference. CN‐LSM sits at an ideological middle ground between mass univariate voxelwise approaches characteristic of conventional LSM methods (i.e., examining many independent, simple models) and more recent advancements in support vector‐based modeling (i.e., simultaneous examination of the whole brain with a multivariate model). CN‐LSM addresses two persistent limitations of conventional LSM. First, it reduces the reliance on per‐voxel statistical significance by shifting the unit of inference from individual voxels to entire predictive maps: candidate regions are screened by resampling, but final inclusion is determined by out‐of‐sample prediction relative to a lesion‐volume benchmark, not by a significance threshold. Its low‐dimensional, two‐coefficient model also keeps each region's contribution directly interpretable—clarity that high‐dimensional SVR‐LSM cannot easily provide. Second, although CN‐LSM yielded lower prediction accuracy than SVR‐LSM on noisy data, its constrained model space led to better recovery of ground truth: within the plausible ensemble, at least one model outperformed SVR‐LSM on F1 in 10/10 (AALCAT) and 8/10 (AICHA) noisy datasets, even though SVR‐LSM achieved lower mean prediction error. CN‐LSM is therefore preferable when the goal is interpretable localization and robust recovery of true regions, whereas SVR‐LSM retains an edge when raw out‐of‐sample prediction is the priority. Examining an ensemble of plausible models across multiple atlases provided converging evidence that increases confidence in a subset of the results.

CN‐LSM is intended to provide an alternative lesion‐symptom mapping approach that is less dependent on inferential statistics based on NHST (Hofman et al. 2021; Simmons et al. 2011; Wicherts et al. 2016; Yarkoni and Westfall 2017), while still maintaining a degree of control over the model in the tradition of conventional quantitative methods in behavioral neuroscience. While data‐driven machine learning approaches have advantages in controlling for false positives arising from model underspecification in conventional linear modeling, the high‐dimensional feature space is difficult to interpret at best and prone to modeling unforeseen confounds at worst. SVR‐LSM requires hyperparameter tuning and other model optimization in a fashion that can be empirically opaque to researchers and increases the degrees of freedom a researcher faces during model construction. We designed CN‐LSM to be focused around model comparison, which limits these degrees of freedom and the overall number of comparisons made (i.e., opportunities for false positives). The relatively tight constraints placed on the model space in CN‐LSM also improve its generalization to new data by providing a clear path for future studies. In SVR‐LSM, it may be difficult to tell the contributions of specific variables to model performance given the high‐dimensional structure of the model.

Although SVR‐LSM has been applied in predictive paradigms, several drawbacks remain. Its abstract tuning parameters lack a theoretical basis and bear an opaque relationship to the data, and a model's inferred significance is generally unrelated to its out‐of‐sample predictions. More fundamentally, a more flexible model can improve prediction by exploiting spurious or nontheoretical structure without better capturing the ground truth—so a better‐predicting model is not guaranteed to be a better‐localizing one. Finally, the focus on a single optimal model leaves potential converging evidence from alternative models unaddressed.

### 6.2 Implications for Localization of Comprehension and Fluency

Both fluency and comprehension are complex, multifaceted constructs; notably, the comprehension measure's yes/no questions and sequential commands engage verbal working memory, which the Dual Stream model (Hickok and Poeppel 2000, 2004, 2007) links to dorsal‐stream production processes. This is relevant for interpreting overlap between the two measures. The seminal VLSM study by (Bates et al. 2003) implicated the insula and arcuate/superior longitudinal fasciculus for fluency and the MTG, parietal association cortex, and dorsolateral prefrontal cortex for comprehension, noting an anterior–posterior contrast; rather than reporting overlap directly, they inferred shared peri‐Sylvian resources from a 0.59 correlation between the two maps' *t*‐values.

Both SVR‐LSM and CN‐LSM reproduced the expected anterior–posterior contrast, but implied different conclusions about shared substrates. SVR‐LSM produced diffuse maps with only a minuscule overlap in white matter underlying the insula (consistent with DeMarco and Turkeltaub 2018), whereas CN‐LSM—constrained by atlas regions, out‐of‐sample prediction, and combinatoric control of parasitic relationships—consistently identified overlapping effects in posterior insula, arcuate fasciculus, and superior longitudinal fasciculus across all three atlas ensembles. This accords with the Dual Stream model's claim that area Spt (near posterior insula) supports sensorimotor integration for tasks engaging verbal working memory. Finally, whereas Bates et al. (2003) emphasized MTG for comprehension, CN‐LSM provided only weak evidence for MTG (3% inclusion) and instead consistently implicated posterior STG, agreeing with historical findings.

### 6.3 Limitations

It is worth noting that there are other implementations of the SVR‐LSM framework (e.g., Yourganov et al. 2018; Zhang et al. 2014) that can and do yield different results when analyzing the same data. For conciseness, we did not address these other variants in the current manuscript; however, initial simulation testing using the default parameter settings for these implementations provided notably divergent results. This limitation of the current study further emphasizes the need for careful interpretation of LSM results, regardless of the mapping algorithm that is used.

Perhaps the most obvious limitation of CN‐LSM is that it did not grossly outperform SVR‐LSM with regard to prediction accuracy in our reported experiments. There are several reasons why we do not consider this to be a true limitation of CN‐LSM. First, CN‐LSM reduces model complexity to a low‐dimensional multivariate model compared to SVR‐LSM's high‐dimensional feature space; in a sense, it is doing more with less. Achieving similar results without modeling any latent patterns in the data identified via multivariate approaches makes CN‐LSM a competitive alternative to SVR‐LSM, closer to conventional VLSM in its methodological design and interpretability. Second, it has been shown that meta‐analysis across disparate methodological approaches yields results that best approximate ground truth (Botvinik‐Nezer et al. 2020). At a common ROI resolution, the CN‐LSM ensemble inclusion rate significantly discriminated ground‐truth regions from spurious ones (AUC = 0.67–0.75), validating it as an indicator statistic in the same sense as SVR‐LSM's established voxel‐coverage statistic, despite resting on different assumptions. That two independently derived indicators converge on the same regions is precisely the kind of cross‐method agreement that best approximates ground truth. Thus, researchers aiming to best describe the relationship between a given behavior and patterns of lesion damage should synthesize results across multiple mapping paradigms, allowing CN‐LSM and SVR‐LSM to coexist as complementary approaches. On the one hand, we are adding to the proliferation of available methods, but on the other hand, we seek to improve consistency among those who adopt the new approach and provide an alternative, equally valid viewpoint to complement the results of those who do not.

The highly constrained nature of model construction in CN‐LSM introduces a few limitations. Unlike other multivariate approaches, CN‐LSM is designed to investigate a single behavior's relationship to the lesion data at a time and is thus limited in its ability to describe interactions among multiple behaviors. We encourage researchers to carefully consider their research goals when selecting a modeling technique (CN‐LSM or otherwise) for use in a study. Another limitation of CN‐LSM comes from its fundamentally atlas‐based nature. Because damage to brain regions is linked to a researcher‐specified atlas, improper selection of an atlas by the researcher may obfuscate the true anatomy of the investigated brain‐behavior relationship. This also, in theory, limits the upper bound on spatial resolution, as individual voxels are not modeled in the same way they are in VLSM and SVR‐LSM. This is an intentional choice on our part to avoid methodological issues with multiple comparisons. Similarly, the decision to label atlas regions as either “critical” or “non‐critical” may obfuscate interactions between brain regions or other forms of graded brain‐behavior relationships. We include extensive information about ensemble prediction (i.e., the set of models that significantly outperform a bivariate lesion volume control model) to enable researchers to investigate the interactions of individual regions in more detail. For example, a high degree of co‐occurrence between two specific regions in the ensemble network could imply that an atlas that differently carves up the shared space of these regions could result in a better prediction of behavior. Lastly, CN‐LSM can be resource‐intensive when running on local hardware because of the exponential growth in evaluated networks as the number of candidate regions increases. It is possible that software optimization in the form of parallelization or multiprocessing could speed up compute times, something we are looking into.

### 6.4 Future Directions

On the methodological side, the immediate next step in optimizing CN‐LSM as a mapping technique is validating its usage in neurodegeneration‐symptom mapping, as neurodegenerative patients are a complementary pathology to stroke and can help disentangle vasculature‐related confounds from true brain‐behavior relationships (Sperber 2020). Expanding the mapping process in several dimensions (e.g., model multiple behaviors, compare across multiple atlases, use a nonlinear technique to calculate prediction error) offer appealing avenues of future development, but we emphasize caution here as simplification of the methodological design is an explicit goal of CN‐LSM and a more sophisticated mapping may run counter to that.

### 6.5 Software Release

**FIGURE 7:**
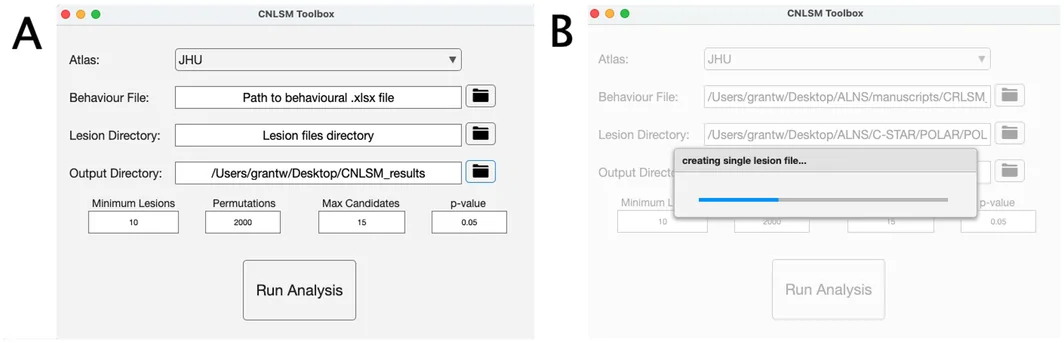
Screenshots of the GUI for the CN‐LSM Toolbox in MATLAB. (A) The paths and parameters are set in the main window. (B) Progress bars update the user about the current step.

To promote reproducibility and adoption, we are releasing CN‐LSM as open‐source software with comprehensive documentation and example datasets. We have developed two MATLAB packages, one using command line interface and the other using a graphical user interface (GUI; Figure 7). We have also developed a Python package with command line interface.

Software runtime was tested on an Apple MacBook Pro with Apple Silicon M5 Max chip, 128GB of RAM, macOS Tahoe 26.5.1, and MATLAB 2026a. The SVR‐DT analysis for comprehension and fluency took 198.7 s. CN‐LSM with the AICHA atlas (i.e., the atlas with the smallest and most numerous regions leading to the greatest computation time) took 114.1 s using the CLI and 120.3 s using the GUI.

## 7 Conclusion

CN‐LSM is a theoretically and methodologically motivated approach to lesion‐symptom mapping that addresses key concerns with currently popular techniques. CN‐LSM greatly reduces the number of comparisons made in voxelwise mass‐univariate approaches and emphasizes prediction and partial correlations over conventional inferential *p*‐values. CN‐LSM also offers comparable predictive accuracy to high‐dimensional multivariate mapping approaches while offering the benefits to interpretability that come from a less complex model design. Importantly, we found that the model never achieved optimal ground‐truth recovery while achieving optimal prediction accuracy. In sum, CN‐LSM offers a unique combination of methodological rigor and minimal researcher degrees of freedom that yield several advantages over alternative approaches: First, it ensures greater robustness to analytical decisions that should be—but often are not—inconsequential to the final results. Second, it provides a holistic quantification of uncertainty across the entire statistical map rather than treating each voxel or region in isolation. Third, it explicitly accommodates the possibility of multiple plausible explanations for observed data, thereby reducing the risk of false negatives while preserving stringent control over false positive rates. The CN‐LSM method provides an expanded view of the behavioral consequences of stroke lesions, productively adding to the toolbox available for neuroimaging analysis.

## Author Contributions

Conceptualization: Grant M. Walker. Methodology: Grant M. Walker. Software: Grant M. Walker. G. Lynn Kurteff. A. Hussain. Formal analysis: Grant M. Walker. Investigation/data collection: Julius Fridriksson (POLAR trial). Resources: Julius Fridriksson, Leonardo Bonilha, Gregory Hickok. Data curation: Julius Fridriksson, Leonardo Bonilha, and Grant M. Walker. Writing – original Draft: Grant M. Walker. Writing – review and editing: Grant M. Walker, G. Lynn Kurteff, A. Hussain, C. Rorden, Leonardo Bonilha, Julius Fridriksson, and Gregory Hickok. Visualization: Grant M. Walker and G. Lynn Kurteff. Supervision: Grant M. Walker, Gregory Hickok, and Julius Fridriksson. Funding acquisition: Gregory Hickok, Leonardo Bonilha, and Julius Fridriksson.

## Funding

This work was supported by National Institute on Deafness and Other Communication Disorders (P50 DC014664).

## Ethics Statement

This study was conducted in accordance with the Declaration of Helsinki. Data were collected as part of the POLAR clinical trial, which was approved by the institutional review board of the University of South Carolina (Pro00053559). All participants provided written informed consent prior to participation. See Spell et al. (2020) and Kristinsson et al. (2023) for full protocol details.

## Conflicts of Interest

The authors declare no conflicts of interest.

## Supporting information


**Table S1:** hbm70644‐sup‐0001‐TableS1.xlsx.

## Data Availability

The neuroimaging and behavioral data used in this study are derived from the POLAR clinical trial (Kristinsson et al. 2023) and are available to qualified researchers upon reasonable request to the corresponding author. The CN‐LSM software (MATLAB CLI, MATLAB GUI, and Python packages) is openly available at: https://github.com/gmwalker‐phd/cnlsm.git.
